# Soil organic carbon stocks increased across the tide-induced salinity transect in restored mangrove region

**DOI:** 10.1038/s41598-023-45411-w

**Published:** 2023-11-13

**Authors:** Huimin Zou, Xianglan Li, Sen Li, Zhe Xu, Zhitong Yu, Houcai Cai, Wandong Chen, Xiaopin Ni, Erwei Wu, Guihou Zeng

**Affiliations:** 1 College of Global Change and Earth System Science, Faculty of Geographical Science, State Key Laboratory of Remote Sensing Science, Beijing Normal University, Beijing, 100875 People’s Republic of China; 2 National Ocean Technology Center, Tianjin, 300112 People’s Republic of China; 3 Qian Xuesen Laboratory of Space Technology, China Academy of Space Technology, Beijing, 100094 People’s Republic of China; 4 Nanji Islands National Marine Nature Reserve Administration, Wenzhou, 330326 People’s Republic of China

**Keywords:** Wetlands ecology, Restoration ecology, Biogeochemistry, Ecology

## Abstract

Blue carbon in mangrove ecosystems contributes significantly to the global carbon cycle. However, large uncertainties maintain in the soil organic carbon (SOC) storage throughout the tide-induced salinity and alkalinity transect in the mangrove restoration region in Southern China. Total 125 soil samples were obtained to detect the SOC content and physicochemical properties. The mean SOC content of each layer ranged from 6.82 to 7.86 g kg^−1^, while the SOC density ranged from 2.99 to 11.41 kg m^−2^, increasing with soil depths. From different land covers in the study region, the SOC content varied from 4.63 to 9.71 g kg^−1^, increasing across the salinity and alkalinity transect, while the SOC density fluctuated from 3.01 kg m^−2^ in mudflats to 10.05 kg m^−2^ in mangrove forests. SOC concentration was favorably linked with total nitrogen (r = 0.95), and total phosphorus (r = 0.74), and negatively correlated with Cl^−^ (r = − 0.95), electrical conductivity (r = − 0.24), and total dissolved solids (r = − 0.08). There were significant logarithmic relationships between SOC content and the concentrations of clay (r = 0.76), fine silt (r = 0.81), medium silt (r = − 0.82), and coarse silt (r = − 0.78). The spatial patterns of SOC concentration were notably affected by soil texture, physicochemical properties, and land-cover type, providing essential reference for future investigations of blue carbon budget in restored mangrove forests.

## Introduction

China stated that it will seek to reach peak CO_2_ emissions by 2030 and carbon neutrality by 2060 during the public conversation of the United Nations General Committee's 75th session in September 2020^1^. To achieve the commitment, China should halt the destructive activities on coastal wetlands, conserve the function and structure of existing coastal aquatic systems, rehabilitate and strengthen their blue carbon role, and reap the benefits of carbon sequestration while conserving the environment^2^. China aggressively and persistently encourages restoration and construction of coastal wetland ecosystem, e.g., mangrove restoration^3^.

Mangrove ecosystems, one of the most carbon-rich inter-tidal communities in tropical and subtropical areas, have a higher CO_2_ deposition rate and low methane emissions, making them crucial to the global carbon cycle and climate change mitigation^4,5^. Although mangrove ecosystems make up just about 0.5% of coastal land and < 0.1% of the total land area, they generate 10–15% of coastal sediment carbon storage^4–6^. Besides, organic carbon from plant and soil within the blue carbon ecosystems has already been identified as a potentially important climate mitigation option^7^. Therefore, it is essential to accurately evaluate the carbon stocks in mangrove ecosystems for better understanding of the global carbon cycle.

Previous studies have demonstrated that a large proportion (~ 60%–90%) of mangrove ecosystem carbon was accumulated in the soil^8–12^. Carbon storage in 12 mangrove forests in Hainan was 16.81 × 10^4^ Mg, with soil accounting for about 80%^13^. The carbon stock increased from the seaward zone to the landward zone in Micronesian mangrove forests, among which about 70% of ecosystem carbon stocks was fixed in soil^14^. Many factors had been reported affecting the dispersion of soil organic carbon (SOC) within mangrove ecosystems, including climatic factors, soil properties (e.g. nutrient availability, bulk density, and C:N ratio), and hydrodynamic processes^15–18^. Sanders, Maher^19^ showed that ecosystem carbon storage was mainly influenced by rainfall in Australia and the Indo-Pacific region. On a global scale, Jardine and Siikamäki^20^ showed that the SOC concentration in mangroves was affected by temperature. Physical protection of carbon in soil aggregates was also an important mechanism for carbon sequestration^21^. Soil physical qualities (e.g. clay and silt concentration) were considered to protect organic materials from decomposer organisms^21^. Particles < 20 μm were essential for retaining the SOC in Baijiang soils and black soils in Jilin province^22^. In addition, a close relationship had been demonstrated among both SOC and the proportion of particle (< 2 μm) in the black soils, and a similar relationship had been found with both SOC and the proportion of fraction (2–20 μm) in the Baijiang soils^22^. These researches demonstrated the necessity of studying the SOC and the complexity of the factors controlling SOC distribution in mangrove forests.

Soil carbon in mangrove ecosystems is either autochthonous (originating from native mangrove generation) or allochthonous (originating from outside via streams)^23^. Mangrove forest is susceptible to recurrent floods and tide exposure, resulting in salt buildup and leaching. SOC stock was also highly negatively linked with tidal range^24^. Because organic carbon is less likely to be washed out of mangrove sediments under micro-tidal conditions, they can store more SOC and hence function as effective carbon sinks^25^. However, it is still unclear how tidal hydrological mechanisms and draining and soaking patterns affect carbon transmission and retention in mangrove ecosystems.

Although mangrove forests store much more carbon per unit area than in other ecological systems^26^, they are suffering from severe deforestation and face an uncertain future because of human-caused changes^5,9,27–29^. Restoration programs had thus been carried out worldwide to offset for the losing of mangrove^30–32^. Natural and restored mangrove forests are primarily found along China's southeast shores in humid tropical and subtropical climates^33^. Zhejiang province represented the northernmost boundary of mangrove planting in China, however, little information is currently available regarding the SOC distribution in restored mangrove forests. It is urgent to carry out field observations on SOC accumulation in restored mangrove forests in South China.

We hypothesize that tide-induced salinity and alkalinity in mangrove forest have an effect on the carbon cycling and carbon deposition. By quantifying the dispersion of SOC concentration, bulk density, and SOC density in soil profiles across different land covers from lower to upper intertidal area, and investigating the relationships between SOC and soil physiochemical properties, we tested the hypotheses (1) that the SOC content increase from the low to high intertidal zone, increasing across the tide-induced salinity and alkalinity transect, and (2) the SOC concentration and distribution characteristics were significantly affected by soil texture, tidal hydrodynamic processes, and land cover.

## Materials and methods

### Study area

**Figure 1:**
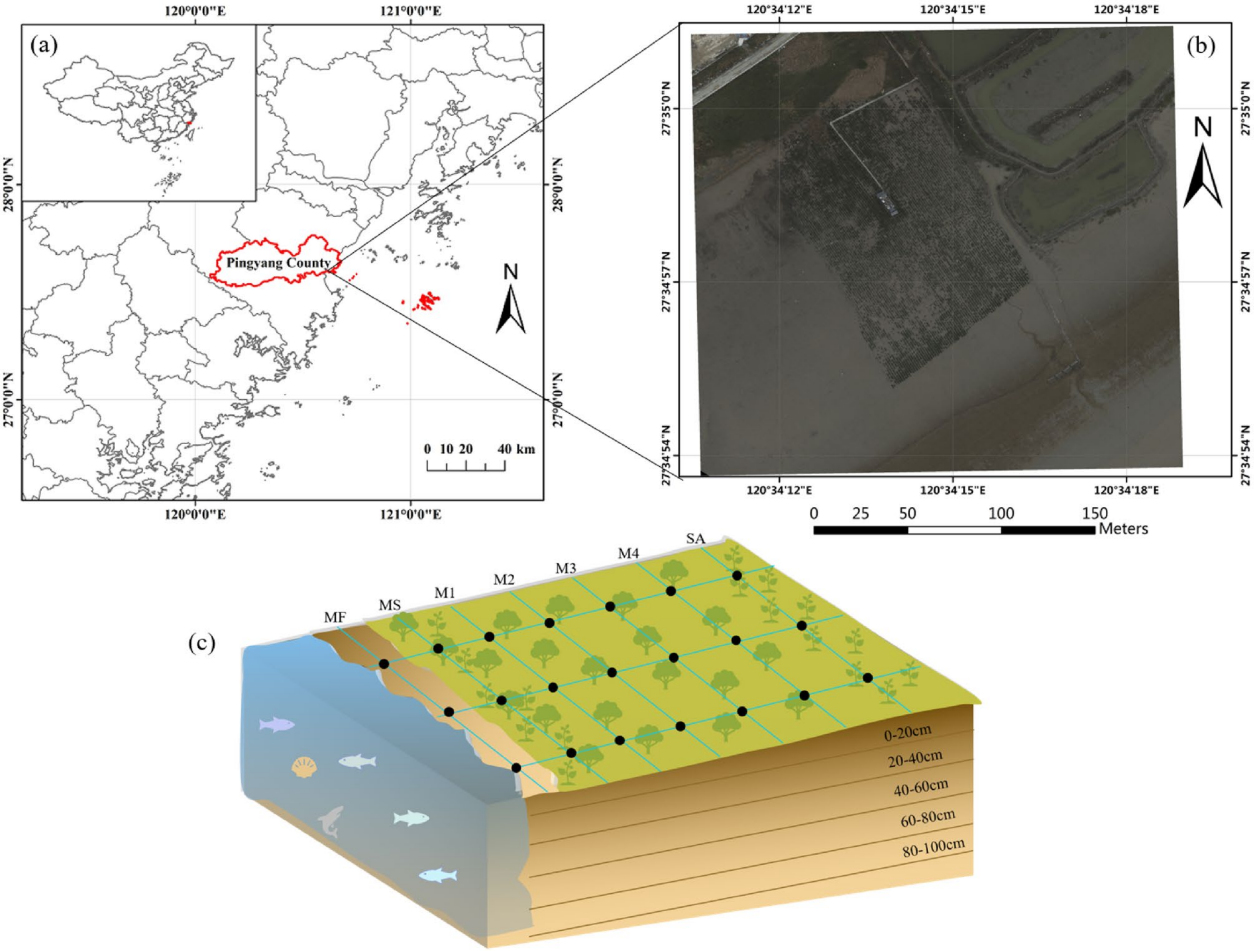
(**a**, **b**) Location of mangrove restoration area in the coastal zone of the Aojiang estuary in Pingyang County, Zhejiang province, China. (**c**) The sampling points in restored mangrove forest in the coastal zone of the Aojiang estuary, which was divided into unvegetated mudflat (MF), mixed area of mangrove and *Spartina alterniflora* (MS), four mangrove communities (M1, M2, M3, M4 from onshore area to offshore area, respectively), and *Spartina alterniflora* (SA). The depth levels for sediment cores were 0–20, 20–40, 40–60, 60–80, and 80–100 cm, respectively.

The restored mangrove ecosystem is located at Aojiang Town, Pingyang County, Zhejiang Province, China (27°18′N, 120°30′E) (Fig. 1a,b). This area is characterized by a subtropical marine monsoon climate, with an annual mean temperature of 18.3 °C and annual precipitation of approximately 1784 mm. The lowest and highest temperatures throughout the year appear in February and August, respectively. This area is affected by continuous rainy weather, with potentially higher rainfall from March to June, and the typhoon period occurs from July to September. The tide is an irregular semi diurnal tide. The maximum tide range occurs twice in the beginning and the middle of the lunar month, and the maximum tide range can reach 603 cm. The mangrove (*Kandelia obovate*) was planted in 2014 on land previously occupied by *Spartina alterniflora*. Various land covers occurred after mangrove plantation in our study area, i.e., unvegetated mudflat (MF), mixed area of mangrove and Spartina alterniflora (MS), four mangrove communities (M1, M2, M3, M4 from onshore area to offshore area, respectively), and Spartina alterniflora (SA).

### Soil sampling and analyses

We collected samples along three transects across the Aojiang estuary and seven parallel to the Aojiang River to assess the carbon stock capacity of different land cover in August 2018 (Fig. 1c). We separated the study area into seven communities with interval of 20 m, including unvegetated mudflat (MF), mixed area of mangrove and *Spartina alterniflora* (MS), *Spartina alterniflora* (SA), and four mangrove communities such as M1, M2, M3, and M4, respectively, across the tide-induced salinity and alkalinity transect from the offshore to the onshore area. Three core samples were collected and averaged for each point using an open-face stainless-steel soil sampler with an internal diameter of 3.5 cm and length of 1 m. The depth levels for sediment cores were 0–20, 20–40, 40–60, 60–80, and 80–100 cm, respectively. All sediment samples were kept in metal specimen boxes and weighed to the nearest 0.1 g to determine bulk density, after which they were packed in clean, dry, labeled plastic bags. To eliminate any pieces of wood or stones, the samples were air-dried, mixed, and filtered using a 2-mm mesh sieve. For each interval, the bulk density was computed by dividing the dry weight by the sample volume (calculated from the boxes) and soil water content, as a percentage.

The impact of environmental factors on SOC were tested according to Guo et al.^34,35^. The potassium dichromate oxidation-heating method was used to detect SOC^36^. A pHS-2C pH meter (Shanghai Youyi Instrument Co., Ltd., Shanghai, China) was used to test the pH of the soil using a soil: water (1:5) mixture. A DDS-307 conductivity meter was used to detect electronic conductivity (EC) (Shanghai Precision Instrument Co., Ltd., Shanghai, China). The concentration of total nitrogen (TN) was measured using six linked nitrogen distillers via the perchloric acid-sulfuric acid digestion method^37^. The concentration of total phosphorus (TP) was measured by the Mo-Sb colorimetric method^34^ using a UVmini-1240 spectrophotometer (Shimadzu Instruments (Suzhou) Co., Ltd., China), and the concentration of total potassium (TK) was measured by acid solution-flame photometry using an FP6410 flame photometer (Shanghai Jingke Instrument Factory, Shanghai, China). Total dissolved solids (TDS) was measured by the residue drying-quality method and the Cl^−^ concentration was measured by silver nitrate titration. According to soil particle size, the soil core was divided into clay (diameter < 2 μm), fine silt (F-Silt, 2 μm < diameter < 16 μm), medium silt (M-Silt, 16 μm < diameter < 32 μm), coarse silt (C-Silt, 32 μm < diameter < 64 μm) and sand (diameter > 64 μm).

### Calculations and statistical analyses

Bulk density and SOC density were determined after removal of wood and stones using the following formulas^35^:

$$\rho_{b} = \frac{m}{{V\left( {1 + \theta_{m} } \right)}}$$

$$SOCD = \sum\limits_{i = 1}^{n} {SOC_{i} \times D_{i} \times \rho_{b} /100}$$

where *ρ*_b_ is bulk density (in g cm^−3^), *m* is the weight of wet soil in the aluminum specimen boxes (in g), *V* is the capacity of the aluminium specimen box (in cm^−3^), *θ*_m_ is the soil water content (in %), *SOCD* is SOC density (in kg m^−2^), *SOC*_*i*_ is SOC content (in g kg^−1^), and *D*_*i*_ is depth (in cm).

Principal components analysis (PCA) is a popular multivariate technique for extracting important information from a dataset composed of several inter-correlated quantitative dependent variables^38^. Pearson's correlation coefficients have been utilized to define the degree and orientation of the linear association between two variables^39^ and were presented using a correlation matrix heatmap describing the linear relationship between two parameters. The variables analyzed by PCA and the coefficient matrix heat map were SOC, pH, EC, TDS, Cl^−^, TN, TP, TK, clay, F-silt, M-silt, C-silt, and sand. All analyses and figures were performed using Python version 3.8.

Across different land cover species, SOC, pH, EC, TDS, Cl^−^, TN, TP, TK, clay, F-silt, M-silt, C-silt, and sand were tested using one-way ANOVA which was performed using SPSS 19.0 (SPSS for Windows, SPSS, Inc.). Differences were regarded as significant when P was less than 0.05. ANOVA analysis was carried out for the mean values ± standard deviation of soil properties, however, no significant difference was found in the results (P > 0.05).

## Results

### Soil physicochemical properties

The SOC content ranged from 6.82 ± 1.51 to 7.86 ± 2.18 g kg^−1^ (mean 7.32 ± 1.94 g kg^−1^) depending on the soil depths, with the highest value at 20–40 cm (Table 1). The soil pH did not differ significantly between the levels (mean 8.01 ± 0.12). Soil EC, Cl^−^ concentration, and TDS concentration ranged from 3.86 ± 0.85 to 4.07 ± 0.85 ms cm^−1^, 5.50 ± 1.41 to 5.83 ± 1.28 g kg^−1^, and from 10.85 ± 2.48 to 11.33 ± 2.27 g kg^−1^, respectively. The highest bulk density occurred at 0–20 cm soil layer (1.86 ± 0.59 g cm^−3^) and the lowest at 60–80 cm soil depth (1.55 ± 0.48 g cm^−3^). TN, TP, and TK concentrations were 0.84 ± 0.13 to 0.94 ± 0.21 g kg^−1^, 0.65 ± 0.02 to 0.68 ± 0.05 g kg^−1^, and 19.83 ± 0.74 to 20.28 ± 0.77 g kg^−1^, respectively, at various depths. Clay and F-silt content were greater in the top fractions (0–20 and 20–40 cm) than in the deeper layers (> 40 cm), although M-silt and C-silt content were lower in the 0–20 and 20–40 cm soil depths than in the 40–60 cm, 60–80 cm, and 80–100 cm soil depths, respectively. The content of sand at 80–100 cm soil depth (0.09 ± 0.15%) was significantly lower than that in other soil layers (from 0.15 ± 0.19% to 0.23 ± 0.39%).

**Table 1:** Mean values ± standard deviation of soil organic carbon (SOC), soil pH, total nitrogen (TN), total phosphorus (TP), total potassium (TK), bulk density, electronic conductivity (EC), the concentration of Cl^−^, total dissolved solids (TDS), clay, fine silt (F-silt), medium silt (M-silt), coarse silt (C-silt) and sand at different soil depths.

	SOC content (g kg^−1^)	pH	TN (g kg^−1)^	TP (g kg^−1^)	TK (g kg^−1^)	Bulk density (g cm^−3^)	EC (ms cm^−1^)	Cl^−^ (g kg^−1^)	TDS (g kg^−1^)	Clay (%)	F-silt (%)	M-silt (%)	C-silt (%)	Sand (%)
0–20 cm	7.57 ± 2.47	8.00 ± 0.11	0.93 ± 0.23	0.68 ± 0.05	20.09 ± 1.10	1.86 ± 0.59	4.07 ± 0.85	5.83 ± 1.28	11.33 ± 2.27	15.51 ± 1.99	65.42 ± 5.28	15.50 ± 4.31	3.34 ± 2.61	0.23 ± 0.39
20–40 cm	7.86 ± 2.18	8.00 ± 0.11	0.94 ± 0.21	0.68 ± 0.04	20.28 ± 0.77	1.72 ± 0.63	3.86 ± 0.85	5.50 ± 1.41	10.85 ± 2.48	15.21 ± 1.62	65.49 ± 4.15	15.89 ± 3.79	3.25 ± 1.88	0.15 ± 0.19
40–60 cm	7.43 ± 1.71	8.00 ± 0.12	0.88 ± 0.18	0.66 ± 0.02	20.00 ± 0.86	1.62 ± 0.54	3.98 ± 0.81	5.62 ± 1.38	11.29 ± 2.38	14.84 ± 1.66	64.48 ± 4.48	16.79 ± 3.82	3.69 ± 2.13	0.21 ± 0.37
60–80 cm	6.93 ± 1.41	8.03 ± 0.13	0.84 ± 0.13	0.65 ± 0.02	19.86 ± 0.71	1.55 ± 0.48	3.91 ± 0.72	5.58 ± 1.16	10.96 ± 2.28	14.44 ± 1.21	63.70 ± 3.02	17.74 ± 2.87	3.93 ± 1.31	0.19 ± 0.34
80–100 cm	6.82 ± 1.51	8.03 ± 0.12	0.84 ± 0.15	0.66 ± 0.03	19.83 ± 0.74	1.65 ± 0.52	4.02 ± 0.68	5.73 ± 1.07	11.29 ± 2.00	14.91 ± 1.47	64.43 ± 3.87	16.90 ± 3.41	3.67 ± 1.81	0.09 ± 0.15
Mean	7.32 ± 1.94	8.01 ± 0.12	0.89 ± 0.19	0.67 ± 0.04	20.01 ± 0.86	1.68 ± 0.57	3.97 ± 0.79	5.65 ± 1.27	11.14 ± 2.30	14.98 ± 1.64	64.70 ± 4.27	16.57 ± 3.75	3.58 ± 2.00	0.17 ± 0.30

**Figure 2:**
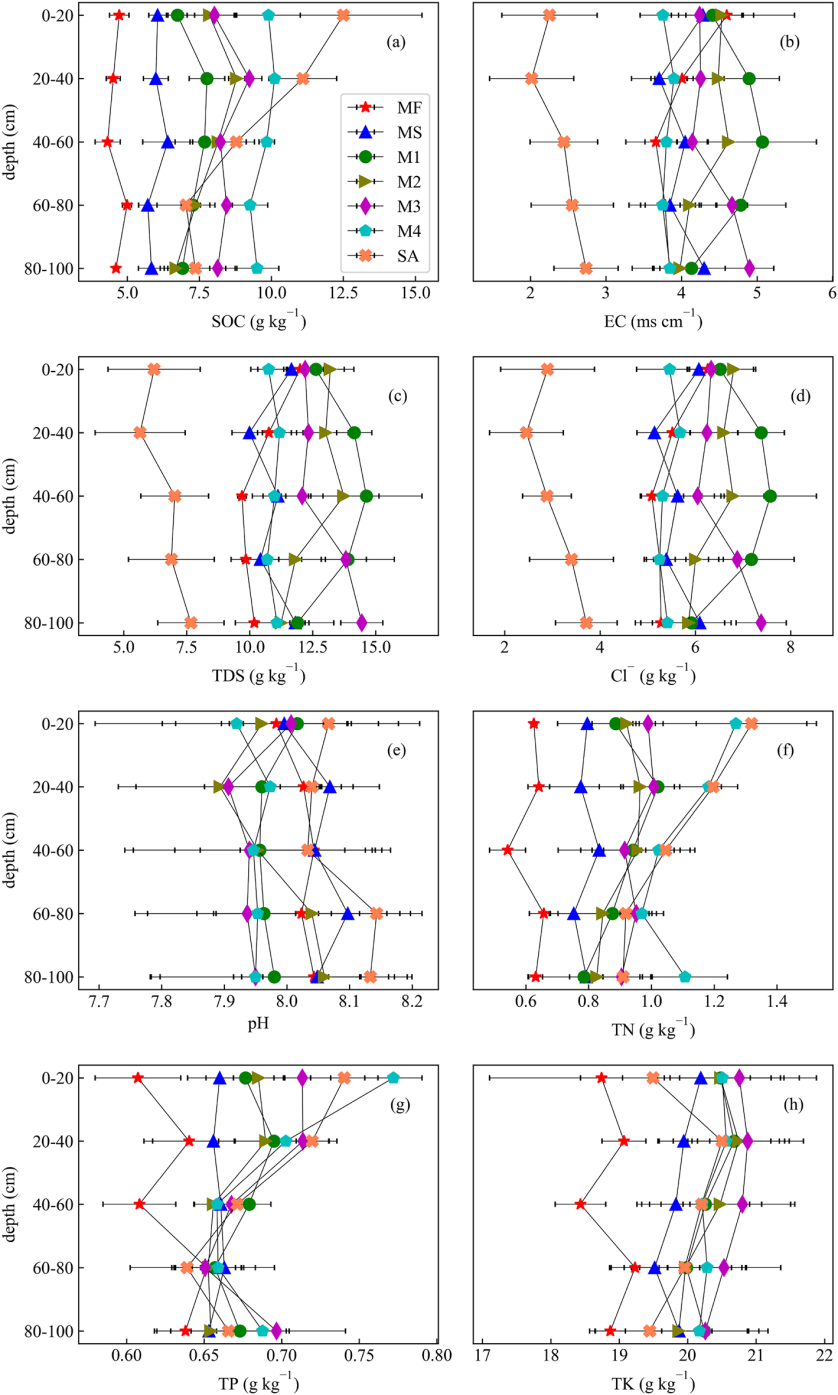
SOC content (**a**), electronic conductivity (EC) (**b**), total dissolved solids (TDS) (**c**), the concentration of Cl^−^ (**d**), pH (**e**), total nitrogen (TN) (**f**), total phosphorus (TP) (**g**), and total potassium (TK) (**h**) in different soil layers across unvegetated mudflat (MF), mixed area of mangrove and *Spartina alterniflora* (MS), four mangrove communities (M1, M2, M3, M4 from onshore area to offshore area, respectively), and *Spartina alterniflora* (SA).

The SOC content in the four mangrove forest regions (M1, M2, M3 and M4) was higher at depths down to 40 cm and then decreased with increasing depth (Fig. 2a), with consistent variations in EC, TDS concentration, and Cl^−^ (Fig. 2b–d). The highest SOC contents were found at a depth of 20–40 cm, in the order M4 (10.12 g kg^−1^) > M3 (9.24 g kg^−1^) > M2 (8.79 g kg^−1^) > M1 (7.77 g kg^−1^), showing significant difference (P < 0.05). The SOC concentration in MS was somewhat greater at 40–60 cm (6.40 g kg^−1^) than at the other layers examined, while the highest SOC in MF (4.99 g kg^−1^) was recorded in 60–80 cm. The SOC in SA declined by about 44% from 0 to 20 cm (12.50 g kg^−1^) to 60–80 cm (7.04 g kg^−1^), and the EC decreased from 2.25 ms cm^−1^ at 0–20 cm soil depth to 2.02 ms cm^−1^ at 20–40 cm, and then increased with soil depth (Fig. 2b). The EC increased slightly in the upper layers (0–60 cm) in M1 and M2, but decreased slightly in the same layers in MF and M3. The tendency of pH in M1, M2 and M3 is consistent, showing a trend of first decreasing and then increasing with increasing depth (Fig. 2e). The concentrations of TN in M4 and SA decreased markedly (P < 0.05) from 1.27 and 1.32 g kg^−1^, respectively, near the surface to 0.97 g kg^−1^ and 0.91 g kg^−1^ at 0–80 cm (Fig. 2f). Interestingly, the lowest value of TN in MF and the highest value in MS were both found at 40–60 cm. The concentrations of TP in all seven land-cover types decreased from 20–40 to 40–60 m soil layers (Fig. 2g). The concentrations of TK increased from 0–20 to 20–40 m soil layers except for mixed area of mangrove and *Spartina alterniflora*. The concentrations of TK in mixed area of mangrove and *Spartina alterniflora* decreased with depth within soil layer of 0–80 cm (Fig. 2h).

### SOC contents in horizontal transect across different land-cover types

**Figure 3:**
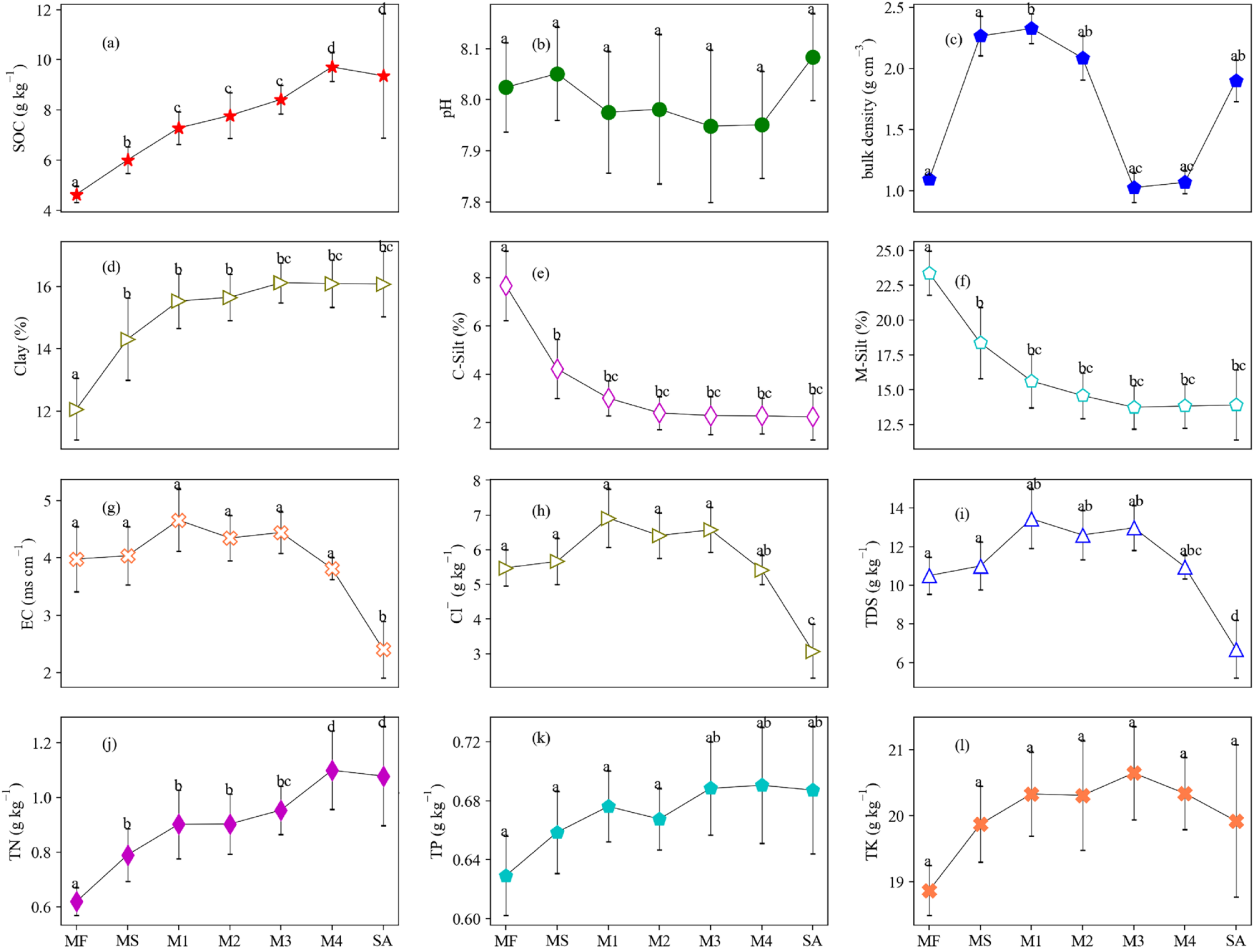
The mean values (standard deviations) of SOC content (**a**), pH (**b**), bulk density (**c**), clay (**d**), C-silt (**e**), M-silt (**f**), EC (**g**), concentration of Cl^−^ (**h**), TDS (**i**), TN (**j**), TP (**k**), and TK (**l**) at top 100 cm.

The SOC content increased with tidal gradient from lower to upper intertidal area, with a slight drop from M4 (9.73 g kg^−1^) to SA (9.36 g kg^−1^) (Fig. 3a). The mean SOC content in the seven land-cover types for the entire soil layer (0–100 cm) decreased in following order: M4 (9.73 g kg^−1^) > SA (9.36 g kg^−1^) > M3 (8.42 g kg^−1^) > M2 (7.77 g kg^−1^) > M1 (7.28 g kg^−1^) > MS (6.00 g kg^−1^) > MF (4.63 g kg^−1^). The differences in mean SOC contents between MF and MS, and M3 and M4 were 1.37 g kg^−1^ and 1.29 g kg^−1^, respectively. Compared with MF, the restored mangrove and sparsely scattered *Spartina alterniflora* increased SOC significantly (P < 0.05). Notably, the SOC content at 0–40 cm depth layer increased in the order of MF < MS < M1 < M2 < M3 < M4 < SA, but it changed at 40–100 cm depth layer due to the drop of SOC content in SA (Figs. 2a and 3a). Soil pH of the sampling points was alkaline (pH > 7, Fig. 3b). Bulk density varied from 1.03 to 2.33 g cm^−3^, with lower bulk densities in MF, M3, and M4 compared with the other stands (Fig. 3c). The concentrations of clay increased (Fig. 3d) from lower to upper intertidal area while the concentration of C-silt (Fig. 3e) and M-silt (Fig. 3f) decreased, especially in the low intertidal zone. EC (Fig. 3g), Cl^−^ (Fig. 3h), and TDS (Fig. 3i) all increased and then decreased from seaward to landward, with the lowest value in SA. TN (Fig. 3j), TP (Fig. 3k), and TK (Fig. 3l) all increased from the low to high intertidal zone, but decreased slightly in M2 and SA.

Comparing with unvegetated mudflat, the colonization of mangrove can increase the SOC content within 60 cm and total nitrogen contents within 40 cm. No significant differences existed between S. apetala and native mangrove communities for the SOC, TN and TP contents (P > 0.05). The restoration of mangrove forests enhanced the soil carbon stock relative to mudflat but significant differences existed only between native mangrove and mudflat. Planted mangroves play important roles in enhancing carbon sequestration and nutrient storage. The mixing native mangrove community should be recommended to enlarge the area of mangrove and enhance the carbon stock capability in the future.

across different land-cover types from mudflats (MF) to *Spartina alterniflora* (SA). Different letters from each line indicate significant differences (p < 0.05) among different land covers according to the one-way ANOVA, which means that if the letters of any two species in the same row are the same, the difference between the index of these two land covers is not significant.

### SOC stocks in restored mangrove forests

**Figure 4:**
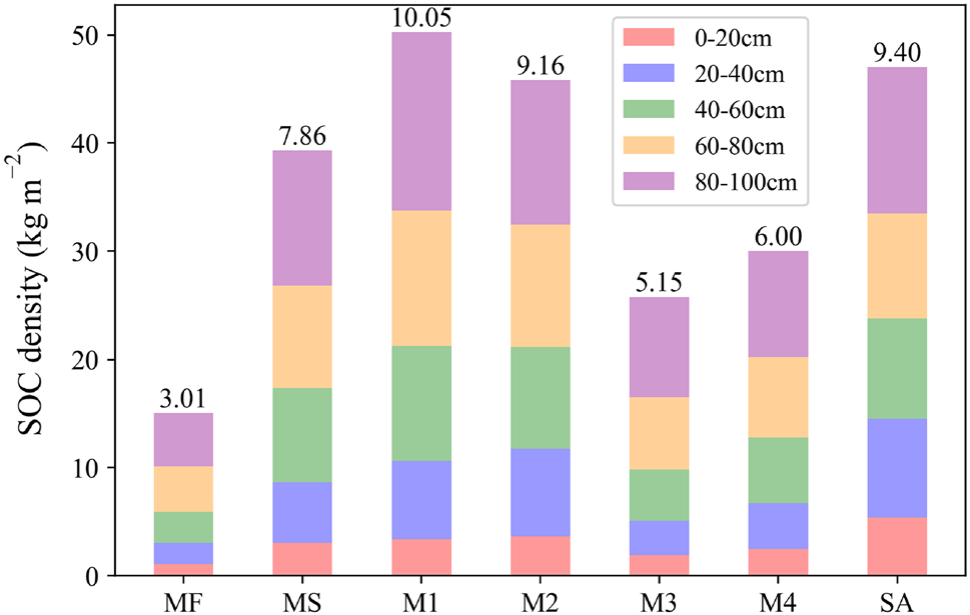
SOC density according to land-cover type at soil depths of 0–100 cm. Five colors from the bottom up represent 0–20 cm, 20–40 cm,40–60 cm, 60–80 cm, and 80–100 cm. Values (in kg m^−2^) above the column represent the average SOC density among five depth layers for different land types.

SOC density showed an increasing trend with increasing soil depth across seven land-cover types, apart from little variation in SA at 20–80 cm soil layer (Fig. 4). Similarly, there was only a slight increase between 40 and 80 cm for MS. The SOC densities ranged from 1.08 to 4.93 kg m^−2^ in MF, 3.05–12.53 kg m^−2^ in MS, 3.36–16.53 kg m^−2^ in M1, 3.63–13.39 kg m^−2^ in M2, 1.90–9.24 kg m^−2^ in M3, 2.49–9.76 kg m^−2^ in M4, and 5.37–13.53 kg m^−2^ in SA. The mean SOC densities for the seven land-cover types throughout the whole soil depth (0–100 cm) were as follow (Fig. 4): M1 (10.05 kg m^−2^) > SA (9.40 kg m^−2^) > M2 (9.16 kg m^−2^) > MS (7.86 kg m^−2^) > M4 (6.00 kg m^−2^) > M3 (5.15 kg m^−2^) > MF (3.01 kg m^−2^). Compared with MF, the restored mangrove significantly increased SOC at the soil depths of 0–100 cm (P < 0.05). With the tidal slope from the lower to upper intertidal zone, the SOC density initially increased, then fell, and finally increased again. The SOC density was substantially greater in vegetation-rich communities than on mudflats. The SOC density (9.40 kg m^−2^) in SA was obviously higher than that in M3 and M4 and similar to that in M1 and M2.

### PCA analysis of SOC and physiochemical properties

**Figure 5:**
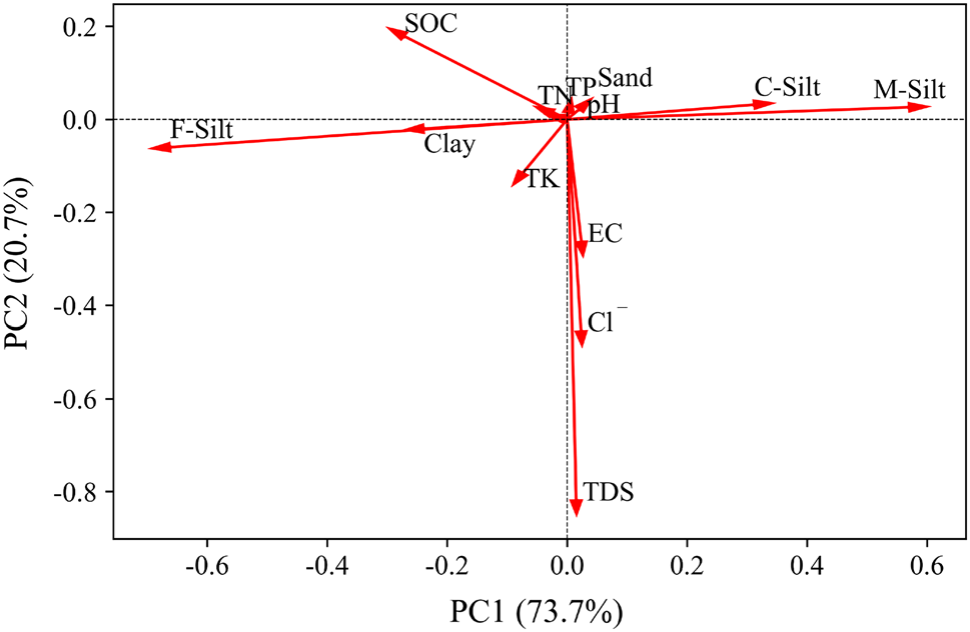
PCA loadings showing correlations of variables (SOC, pH, TN, TP, TK, EC, Cl^−^, TDS, C-silt, M-silt, F-silt, clay, and sand) with principal components PC1 and PC2. Loading value represents strength of correlation with principal component.

PCA captured 94.4% of variations in the first two principal components, with 73.7% explained by PC1 and 20.7% by PC2 (Fig. 5). PC1 was mainly characterized by positive loadings of C-silt and M-silt and negative loadings of SOC, F-silt, clay, and TK. PC2 was mainly characterized by positive loading of SOC and negative loadings of EC, TDS, Cl^−^, and TK. The SOC content was favorably associated with the concentrations of F-silt, TN, TK, and clay, and negatively correlated with the concentrations of M-silt and C-silt, according to the PCA score plot (Fig. 5).

**Figure 6:**
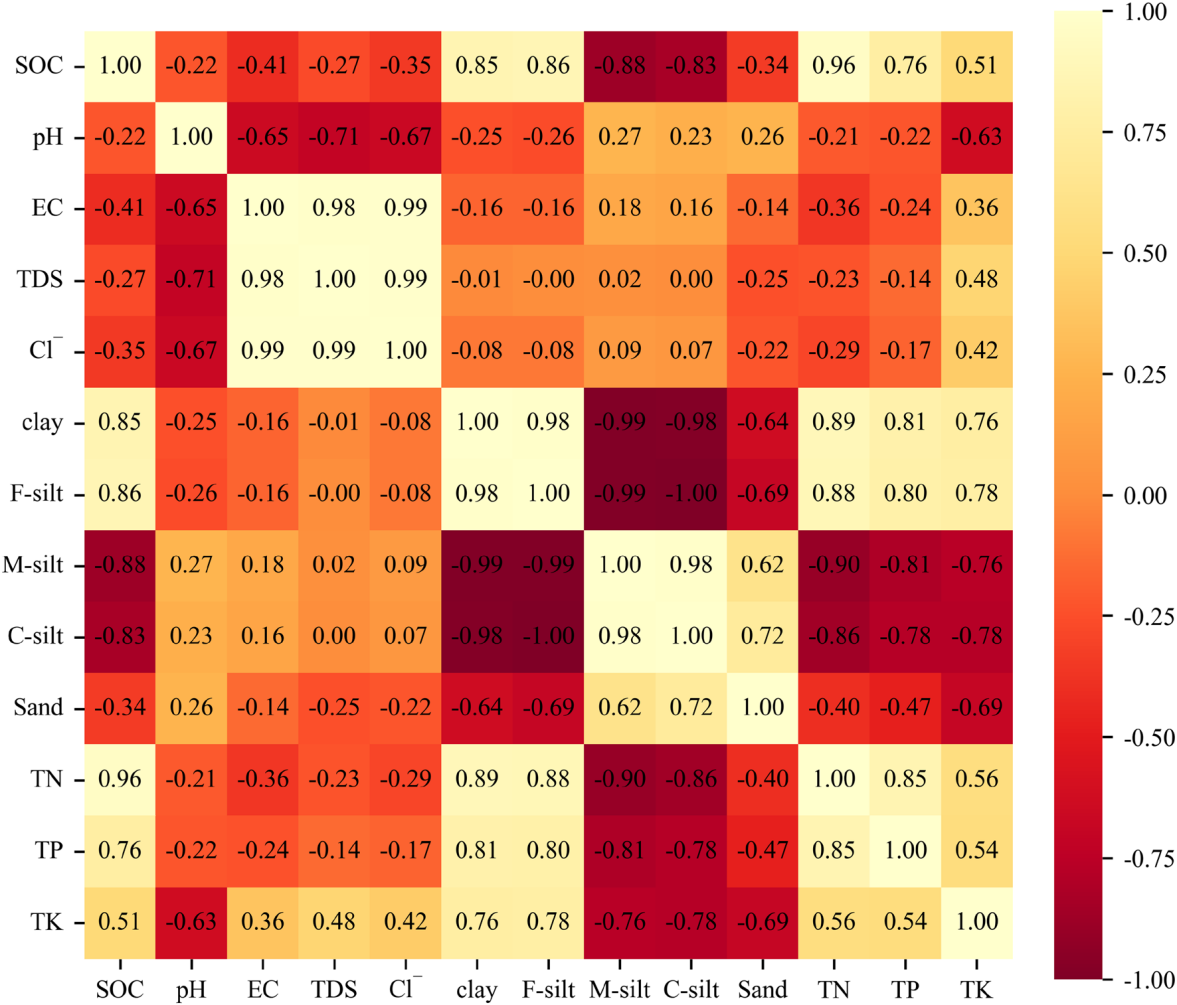
Correlation matrix heatmap generated in Python 3.8 showing Pearson’s correlation coefficients for soil physicochemical properties. Coefficients ranged from − 1 to 1, with − 1 indicating a negative relationship between two variables, 1 indicating a positive relationship, and 0 indicating no relationship.

**Figure 7:**
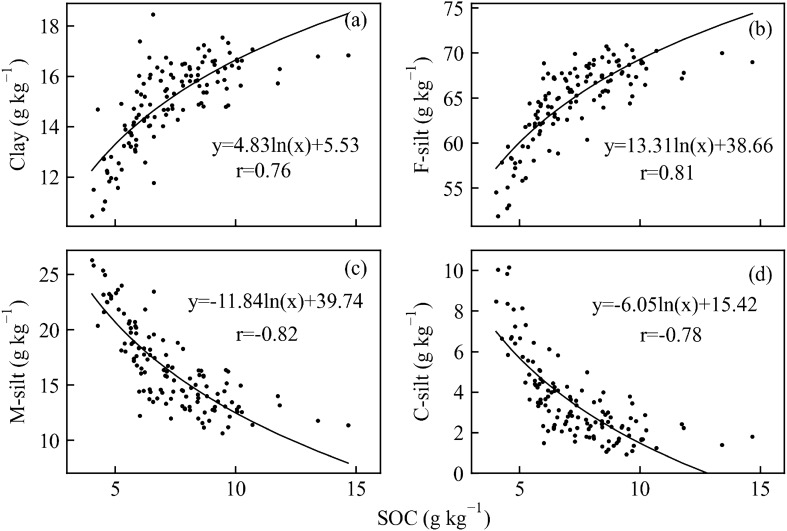
Relationships between SOC and clay (**a**), F-silt (**b**), M-silt (**c**), and C-silt (**d**), P < 0.001.

SOC content showed strong negative relationships with M-silt and C-silt (Pearson’s correlation coefficient − 0.88 to − 0.86) (Fig. 6), strong positive relationships with clay, F-silt, TN, and TP (Pearson’s correlation coefficient 0.74 to 0.87), and negative correlations with sand, EC, TDS, Cl^−^, and pH (Pearson’s correlation coefficient − 0.32 to − 0.08). The concentrations of EC, TDS, and Cl^−^ showed strong positive correlations with each other (Pearson’s correlation coefficient 0.97 to 0.99), and the concentrations of clay and F-silt were strongly negatively related to M-silt and C-silt (Pearson’s correlation coefficient from − 0.99 to − 0.94). We also found strong positive logarithmic relationships between SOC content and clay and F-silt, and strong negative logarithmic relationships between SOC content and M-silt and C-silt (Fig. 7). The r values between SOC content and C-silt, M-silt, F-silt, clay were 0.78, 0.82, 0.81, and 0.76, respectively.

## Discussion

### Comparisons of SOC content with previous studies

The SOC contents (4.63–9.71 g kg^−1^) in this study were similar to those in the other studies^15,40,41^ (Table 2), but lower than those in Dongzhaigang (14.89–20.89 g kg^−1^)^42^, the Jiulong River estuary (15.01–22.25 g kg^−1^)^43^, Vietnam (0.8–21.8 g kg^−1^)^44^, and the southern Saudi Arabian Red Sea coast (28.1–29.3 g kg^−1^)^28^ at lower latitudes. The SOC density in this study (2.99–11.41 kg m^−2^) was comparable to previously reported values (2.6–8.5 kg m^−2^) in the same or similar depth^15,42^, but lower than in the Jiulong River estuary (13.0–17.0 kg m^−2^)^43^. These differences may have been due to differences in biomass associated with climatic conditions, mangrove species, and human activities^45^. Some studies demonstrated that climatic factors were the main determinants of carbon density in mangrove forests^46^. The assessment of SOC concentration may be influenced by soil bulk density, which is an indication of the density of soil^28^. In this study, the mean soil bulk density spanned from 1.55 to 1.86 g cm^−3^ at 0–100 cm soil layer, which was comparable to levels in the Egyptian Red Sea coast (1.40–1.72 g cm^−3^) and the southern Saudi Arabian Red Sea coast (1.5–1.8 g cm^−3^), but higher than those in the Jiulong River estuary (0.82–0.88 g cm^−3^), Leizhou Peninsula (0.93–1.11 g cm^−3^), Dongzhaigang (1.08–1.51 g cm^−3^), Futian Bay (0.93 g cm^−3^, 6yrs-*K.obovata* and 0.79 g cm^−3^, 40 years-*K. obovata*)^9^ and Sundarbans (0.59–0.79 g cm^−3^). We found a negative association among SOC concentration and soil bulk density, which is similar with prior research^47,48^. This negative correlation demonstrated that soil bulk density has an impact on soil porosity, aeration, and construction, which in turn has an impact on SOC content.

**Table 2:** SOC content, bulk density, and SOC density in previous studies.

Dominant species	Location	SOC (g kg^−1^)	Bulk density (g cm^−3^)	SOC density (kg m^−2^)	References
*A. marina*	Egyptian Red Sea Coast (27°24′N, 117°55′E)	4.2–15.5	1.40–1.72	2.6–8.5	^15^
*Kandelia obovata*	Jiulong River Estuary (24°24′N, 117°55′E)	15.01–22.25	0.82–0.88	13.0–17.0	^43^
*Kandelia obovate, Sonneratia apetala*	Futian (22°30′N–22°32′N, 113°56′E–114°03′E)	0.01–0.61	0.71–1.04	3.8–15.2	^9^
*Avicennia marina, Avicennia alba*	Sundarbans (21°32′N–22°40′N, 88°05′E–89°E)	5.14–6.54	0.59–0.79	–	^41^
*A. marina*	Leizhou Peninsula (20°50′N–21°50′N, 109°30′E–110°30′E)	4.9–16.4	0.93–1.11	–	^40^
*Kandelia obovata, *etc.	Vietnam (20°11′N–20°18′N, 106°27′E–106°37′E)	0.8–21.8	–	–	^44^
*A. marina, B. sexangula, *etc.	Dongzhaigang (19°56′N–20°51′N, 110°32′E–110°36′E)	14.89–20.89	1.08–1.51	4.11–5.83	^42^
*Kandelia obovate, Sonneratia apetala, *etc.	Southern China (18°12′N–27°26′N, 108°03′E–121°45′E)	5.39–88.5	–	743.1–7845.0	^33^
*A. marina*	Saudi Arabian Red Sea coast (17°48′N–18°N, 41°40′E–41°53′E)	28.1–29.3	1.5–1.8	16.8–17.2	^28^

In restored mangrove forests, soil carbon sequestration improves with age^49^. Previous studies reported that mature mangrove forests accumulated far more carbon than younger forests^4,45^. The SOC density of 4-yrs restored mangrove forest is 2.99–11.41 kg m^−2^ in this research. Ren, Chen^50^ demonstrated that after 4, 5, 8, and 10 years of restoration, SOC storage rose markedly by 3, 68, 274, and 350 kg m^−2^, respectively, in four *Sonneratia apetala* plantations of different ages in southern China. When compared to the earlier mangroves (17 and 35 years), youthful mangroves (13 years) hold half the carbon sinks rates of older mangroves^49^. Thus, when assessing the potential of mangrove ecosystems to trap carbon, it is crucial to consider their age of the mangrove forests^9,51,52^. Furthermore, short-term mangrove recovery merely increased the top soil carbon pool, with no impact on the subsoil^53^. This information is valuable for carbon offset projects since this affects the carbon retention capacity of freshly planted mangroves and indicates that data from mature mangrove forests cannot be used as direct estimates for carbon dynamics in restored or regenerated mangroves^49^.

### Relationships between SOC and soil physiochemical properties

Several physicochemical properties were correlated with SOC content in this study, indicating that soil texture is vital in SOC accretion. Previous research has found a negative association of SOC content with soil salinity^54,55^, with increased salinity accelerating microbial breakdown, limiting plant growth, and further reducing carbon accumulation^54,56^. This research also discovered a negative association of SOC concentration with salinity. In addition, electronic conductivity and the Cl^−^ concentration were also negatively correlated with SOC content. Although SOC was not significantly correlated with pH in this study, the possible influence of pH should not be ignored; pH could affect the decomposition rate of SOC by inhibiting microbial activity^42,57^, given that most microorganisms prefer to be active when the pH value is around neutral^58^.

The logarithmic relationships between SOC content and particle size are comparable with the study reported by Hassink, Whitmore^59^, demonstrating the proportion of particles < 20 μm is intimately linked to the accumulated carbon content in this proportion in the upper 10 cm. One of the key mechanisms essential to physically protecting SOC is its propensity to bond with clay and silt particles^60^, which represent the most significant carbon sink^61^. Most of the SOC (79%–91%) was thus accumulated in the clay (< 2 μm) and silt (2–20 μm) segments^62^. Fine-textured soils contain more SOC than coarse-textured soils of the same substance source^60,63^. The quantities of carbon affiliated with the silt and clay fractions had been closely correlated with percentages of soil particles in these fragments in both temperate and tropical regions^60^, suggesting that finer sediments may provide a more reactive area that can gather organic carbon, thus protecting organic carbon from remineralization^44^. Besides, mangrove forests have been proved to effectively increase the clay content in soil, which is related to higher carbon accumulation^64^.

Positive relationships were observed in the concentrations of SOC, TN, and TP in the restored mangrove ecosystems (Fig. 5, 6). Mangrove forests, growing in eutrophic waters, have the potential to accumulate SOC, TN, and TP at higher rates than mud flat in this study. *Spartina alterniflora* enhanced the SOC, TN, and TP contents significantly (P < 0.05) comparing with mud flat and mangrove ecosystems (Fig. 3). Both mangrove forests and *Spartina alterniflora* could uptake a tidal nitrogen and phosphorus nutrients to satisfy its requirement for growth (Fig. 3). Previous studies indicated that *Spartina alterniflora* could increase SOC and TN contents by 0.37 and 7.43 times in comparison with the mudflat and native saltmarsh species after 10 years invasion, which demonstrated a great potential for soil carbon and nitrogen accumulation^65–67^. *Spartina alterniflora* significantly enhanced the ecosystem carbon and nitrogen stock in the Yangtze River estuary by altering the ecophysiological process and plant-soil-microbe feedback during vegetation invasion (Liao et al. 2007^68^). Our results indicated that the impact factors on SOC content were complicated, which was necessary to analyze the relationship between SOC and soil properties with the aims to improve the efficiency in blue carbon budget.

### Variations in mangrove SOC across tide and salinity gradients

The SOC content was obviously higher in mangrove forests (7.28–9.73 g kg^−1^) than in the adjacent mudflats (4.63 g kg^−1^), and was higher in landward compared with seaward communities in this study. This result may be attributable to less erosion and a more stable hydrological environment in the landward communities, thus allowing more carbon to be stored in the soil^13^. Most of the soil carbon stock in the top meter is accounted for by autochthonous carbon, such as litter from plants and roots^6,69^, while some is derived from the tide^69,70^. Similar results were found in estuarine mangrove communities in South China^12^ and in the mangroves at Dongzhai Harbor National Natural Reserve^69^. Furthermore, SOC content was substantially greater near the estuary than in the more salinized coastal areas^71^, which is in agreement with our result that SOC content was negatively correlated with TDS. Previous study showed that the SOC in the juvenile mangrove areas is mostly of allochthonous origin (estimated on average at 79%)^72^. And the mangrove areas that are about 50 m from the beach and are not inundated daily. This may explain why the SOC density increased from 3.01 to 10.05 kg m^−2^, and then decreased to 5.15 kg m^−2^ with the tidal gradient from lower to upper intertidal area. This is consistent with the fact that organic carbon levels steadily grow in surficial layers from the seashore up to a certain extent and then fall^24^. The SOC content was greater in SA than other land-cover categories, except for M4, which might have been due to the plant species as well as the tide/salinity gradient. Interestingly, Lunstrum and Chen^9^ reported no significant change in SOC concentrations between *S. apetala* and *K. obovata* over a 6-year period. Further studies are therefore needed to investigate the aboveground and subsurface biomasses of mangrove communities at different locations and in forests of different ages in Zhejiang.

## Conclusions

Our findings provide compelling evidence that restored mangrove forests have superior capacity to sequester carbon in the sediments compared with the mudflat. In the region of mixed mangrove and *Spartina alterniflora* (MS) and single mangrove ecosystems, the SOC content increased initially and subsequently dropped with increasing soil depths. The SOC density rose as soil depths increased. A pattern of increasing SOC content (4.63–9.73 g kg^−1^) along the tide-induced salinity transect was found, while SOC density varied from 3.01 kg m^−2^ on mudflats to 10.05 kg m^−2^ in mangrove forests. SOC content was shown to be favorably connected with TN, TP, and negatively associated with Cl^−^, EC, and TDS. SOC concentration has strong logarithmic associations with clay, F-silt, M-silt, and C-silt. More extensive research is required to increase our comprehension of carbon sinks and its regulating mechanisms in mangrove ecosystems. To meet its vision to support carbon neutrality by 2060, China might also boost scientific studies on coastal habitats, safeguard the functional and structural stability of existing coastal wetlands, prevent catastrophic coastal wetlands activities, reestablish as well as strengthen the "blue carbon" role.

## Data Availability

The datasets generated and analyzed during the current study are not publicly available due to privacy but are available from the corresponding author on reasonable request.
